# Urban tolerance does not protect against population decline in North American birds

**DOI:** 10.1098/rsbl.2023.0507

**Published:** 2024-01-31

**Authors:** Julianna A. Petrenko, Paul R. Martin, Rachel E. Fanelli, Frances Bonier

**Affiliations:** ^1^ Department of Biology, Queen's University, Kingston, Ontario, Canada K7L 3N6; ^2^ Department of Zoology and Physiology, University of Wyoming, Laramie, WY 82071, USA

**Keywords:** anthropogenic effects, biodiversity loss, community science, environmental tolerance, habitat degradation

## Abstract

Population declines of organisms are widespread and severe, but some species' populations have remained stable, or even increased. The reasons some species are less vulnerable to population decline than others are not well understood. Species that tolerate urban environments often have a broader environmental tolerance, which, along with their ability to tolerate one of the most human-modified habitats (i.e. cities), might allow them to persist in the face of diverse anthropogenic challenges. Here, we examined the relationship between urban tolerance and annual population trajectories for 397 North American bird species. Surprisingly, we found that urban tolerance was unrelated to species’ population trajectories. The lack of a relationship between urban tolerance and population trajectories may reflect other factors driving population declines independent of urban tolerance, challenges that are amplified in cities (e.g. climate warming, disease), and other human impacts (e.g. conservation efforts, broad-scale land-use changes) that have benefitted some urban-avoidant species. Overall, our results illustrate that urban tolerance does not protect species against population decline.

## 1. Introduction

Climate change, invasive species, habitat loss and other human-caused challenges degrade ecosystems and threaten biodiversity on a global scale [1]. Ongoing losses of biodiversity are apparent across taxa and have been described as the planet's sixth mass extinction event [2–6]. For example, recent evidence indicates dramatic declines across many bird species [7], including a net loss of almost 3 billion individual birds since the 1970s in North America alone [8]. Understanding why some species are more vulnerable to declines than others is important, but the factors that determine which species are most at risk are not always clear [9–11].

Many species are threatened by anthropogenic habitat alteration and habitat loss, but some species appear able to persist, and sometimes even thrive, in human-altered environments [12]. Urban habitats represent some of the most dramatically human-altered landscapes, and are associated with challenges like altered climate, resources and communities [13,14]. Expanding urbanization also increasingly contributes to global habitat loss [15], particularly in biodiverse coastal and tropical regions [16,17]. Urban tolerance, or the ability to survive and reproduce in cities, varies among species [18,19] and is generally higher in species with broader environmental tolerance and generalist ecologies [20–23]. Thus, urban tolerant species might be less vulnerable to range-wide population declines caused by habitat loss and other anthropogenic challenges because of their broad environmental tolerance, and ability to persist in one of the most human-altered environments. In other words, urban tolerant species, which occur in both urban and non-urban environments, might be protected from population declines due to their ability to cope with a broad array of anthropogenic challenges, including those associated with urbanization. To our knowledge, no study has assessed links between urban tolerance and contemporary population declines.

Here, we address the question: does a species' urban tolerance predict its range-wide population trajectory? We draw on recent research that provides quantitative urban tolerance estimates for North American breeding bird species [18] along with long-term species’ population trajectories [24]. We incorporate several ecological traits into our analyses, to investigate potential context-dependence of relationships between urban tolerance and population trajectories. We predict that more urban-tolerant species would be more resilient to anthropogenic challenges, and therefore would have more stable or even increasing populations, relative to urban-avoidant birds.

## 2. Methods

### (a) Population trends

We derived species-level mean annual trends and variance in these estimates from the North American Breeding Bird Survey, a community-science database which tracks the status and trends of North American bird populations [24]. Species' mean annual trends are estimated by integrating long-term population change and current population size into a hierarchical Bayesian model estimating the species’ range-wide population size through time (see [24] for details). We retrieved mean annual trends (estimated annual per cent change in population size for each species, averaged across 1993–2019) for the contiguous USA and most of Canada.

### (b) Urban tolerance

We obtained species-level estimates of urban tolerance from Fanelli *et al*. [18], who quantified species' tolerance of urban habitats based on range-wide estimates of relative abundance during the breeding season, derived from eBird Status & Trends data [25] and two different geospatial datasets that categorize and quantify urbanization. Specifically, we used a multivariate urban tolerance score derived from a principal component analysis of two of the best-performing, independent urban tolerance estimates [18]: (i) the standardized difference in relative abundance of each species in urban versus non-urban classified habitats, and (ii) the mean nighttime illumination in areas where each species occurred relative to their entire range (including areas of occurrence and absence). We used this multivariate, compressed score because it distinguished 24 known urban-tolerant and urban-avoidant species, and was highly correlated with an independent, coarser estimate of urban tolerance from previous work [26], suggesting that it provided an accurate estimate of urban tolerance across species (see Metadata in [18] for details).

### (c) Ecological traits

We considered several types of ecological trait data to determine if a species’ ecology modifies the relationship between urban tolerance and population trajectories. We derived primary diet data from EltonTraits, a database reporting key traits for an extensive group of birds and mammals [27]. We condensed the original diet categories used by EltonTraits into five categories: carnivore (including piscivores and scavengers), herbivore/granivore, frugivore/nectivore, invertebrate diet and omnivore (when no other diet category comprised 60% or more of a species' diet). We derived population size estimates (the estimated number of individuals of each species found in the continental USA and Canada in 2015), breeding biome (aridlands, arctic tundra, boreal forests, coasts, eastern forests, grasslands, western forests, wetlands, forest generalist, habitat generalist or introduced) and migratory status (resident, migratory or mixed for species with both migratory and sedentary populations) from Rosenberg *et al*. [8]. We included population size in our analyses because larger annual per cent changes might be more common in smaller versus larger populations, and because population size might modulate the relationship between urban tolerance and population trajectories. We compiled body mass data from published sources, using the most recent data that were derived from individuals sampled in the USA or Canada [28–30].

The final dataset includes 397 species (60 families, 19 orders) of birds with complete mean annual trend, urban tolerance and ecological trait data.

### (d) Statistical analyses

We conducted all analyses using R version 4.3.0 [31]. We evaluated the relationship between urban tolerance and species’ trajectories using linear mixed-effects models (LMM, with the function *lme* in the R package *nlme*; version 3.1–160, [32]) with mean annual trends as the response variable and urban tolerance estimates as a fixed effect. To consider potential modifying effects of ecological traits, we also included primary diet, population size, breeding biome, migratory status and body mass, as well as each trait's two-way interaction with urban tolerance as additional fixed effects in the global model. We included taxonomic family as a random intercept, to control for phylogenetic non-independence of species. To improve model fit and meet model assumptions, we arcsin transformed annual trend, log10-transformed population size and body mass estimates, and square-root transformed urban tolerance scores (+2.05, to make all values positive prior to transformation). See electronic supplementary material for description of assessments of model fit.

We used an information theoretic approach, using the *dredge* function in the package *MuMIn* (version 1.47.1, [33]), to compare all recombinant versions of the fixed effects included in the global model, including a null model. We present results from model selection as well as results of the best-performing model (lowest Akaike's information criterion corrected for small sample size, AICc) that retained urban tolerance to test the hypothesis that urban tolerance predicts species' mean annual trends. We conducted three additional analyses as supplements to the main analyses. First, we repeated the highest-ranked model that retained urban tolerance identified above using Bayesian phylogenetic mixed-effects models (BPMM) with the *MCMCglmm* function in the package of the same name (version 2.34, [34]), including a species phylogeny as a random effect (electronic supplementary material, figure S1). Second, because the annual trends were model estimations and subject to uncertainty, we repeated the main analysis including model selection, but with a term included in all models to weight the analysis by the inverse of the 95% confidence intervals for the annual trends, such that trends associated with greater uncertainty would be discounted relative to trends with less uncertainty. Finally, we repeated the BPMM analysis of the highest-ranked model that retained urban tolerance identified in our second supplemental analysis (which was the same as the model selected in the main analysis), using the same methods as described above, but including the 95% confidence interval of the annual trends in a term that accounts for measurement error (*mev* in *MCMCglmm*).

## 3. Results

**Figure 1.**
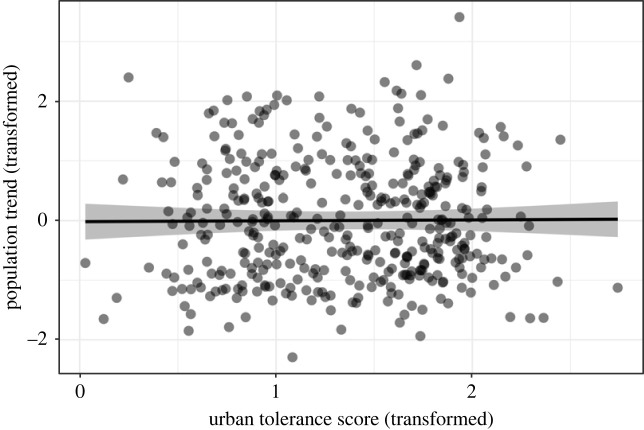
Urban tolerance does not predict population trends in 397 North American bird species. Each point represents one species, the line illustrates the model-predicted relationship between urban tolerance (*x*-axis, square-root transformed) and population trends (mean annual per cent change, arcsin transformed), and the shaded area shows the 95% confidence interval. Darker circles reflect overlapping datapoints. Model results are illustrated here, controlling for a relationship with body mass (see electronic supplementary material, figure S2 for a plot of back-transformed data).

Urban tolerance did not predict variation in annual population trends among our 397 focal species of North American birds. Urban tolerance was not retained in the highest-ranked model to explain variation among species in mean annual trends (electronic supplementary material, table S1). The top model only retained body mass; larger birds had higher mean annual trends (LMM, log_10_ body mass: *β* = 0.35 ± 0.09, d.f. = 336, *t* = 3.91, *p* < 0.001), indicating their populations were more stable or increasing relative to smaller birds. The second-ranked model (ΔAICc = 0.84) included body mass and urban tolerance, which was unrelated to mean annual trend (LMM, square-root urban tolerance: *β* = 0.01 ± 0.10, d.f. = 335, *t* = 0.15, *p* = 0.88; figure 1). Bayesian models controlling for phylogeny produced similar results, although the relationship between body mass and mean annual trend was less evident (BPMM, square-root urban tolerance: *β* = 0.01, pMCMC = 0.95; log_10_ body mass: *β* = 0.18, pMCMC = 0.13). Analyses weighted by the inverse of the 95% confidence interval of the annual trends identified the same top two models as our main analysis, with similar results (LMM, square-root urban tolerance: *β* = −0.05 ± 0.11, d.f. = 335, *t* = −0.48, *p* = 0.63; log_10_ body mass *β* = 0.32 ± 0.10, d.f. = 335, *t* = 3.24, *p* = 0.001; electronic supplementary material, figure S3 and table S2). The Bayesian phylogenetic model that included a measurement error term also produced similar results to our main analysis (BPMM, square-root urban tolerance: *β* = −0.04, pMCMC = 0.71; log_10_ body mass: *β* = 0.24, pMCMC = 0.03).

## 4. Discussion

Our study provides the first test of the hypothesis that urban tolerance protects species from population declines caused by anthropogenic challenges. We assessed the relationship using range-wide population trajectories for 397 North American bird species paired with novel species-level urban tolerance estimates, predicting urban-tolerant species would be less vulnerable to decline. Surprisingly, results reveal urban-tolerant bird species were as vulnerable to decline as urban-avoidant species (figure 1).

Urban-tolerant species appear to be vulnerable to decline because of other, non-urban challenges that impact populations. For example, chimney swift (*Chaetura pelagica*) is widespread in cities, using chimneys as nesting and roosting sites [35]. Nonetheless, chimney swift populations, along with those of many other aerial insectivores, have declined dramatically [36]; their shared reliance on flying insects suggests that reduced access to food might be an important cause of declines across species, regardless of their urban tolerance [37–39]. Similarly, house finch (*Haemorhous mexicanus*) is common in cities but has shown recent, widespread population declines resulting from a bacterial disease [40]. Disease transmission is facilitated by bird feeders [41], illustrating how urban environments could amplify the effects of other challenges, with consequences for populations. Similar synergistic interactions, such as extreme heat resulting from the interaction between cities and climate warming [42], are likely to present further obstacles for urban-tolerant species. Results from North American birds parallel previous research documenting the declines of some European urban-tolerant birds in response to climatic and land use changes [43], in addition to the challenges of urban habitats themselves [44].

While some urban-tolerant species have shown population increases with increasing urbanization, some urban-avoidant species have benefited from conservation efforts and broad-scale changes in land use. For example, the banning of the pesticide DDT (dichloro-diphenyl-trichloroethane), which caused massive declines in many top predators, and the protection of predatory birds from human persecution, have led to recent increases in populations of less urban-tolerant birds, like bald eagle (*Haliaeetus leucocephalus*) [45,46]. Similarly, regrowth of forests on abandoned farmland in parts of North America has fuelled an increase in populations of some urban-avoidant forest birds, like blue-headed vireo (*Vireo solitarius*) [47–49]. Altogether, the diverse impacts of humans on bird populations appear to underlie the lack of a relationship between urban tolerance and population trajectories that we found in our study.

Overall, our study reveals that urban tolerance does not protect species against population decline. The surprising vulnerability of urban-tolerant birds points to the severity of the ongoing threats to biodiversity, and illustrates that the traits that allow some species to thrive in cities are not sufficient to buffer against these threats. As human-induced changes to the biosphere continue, even the species that are best suited to cope with anthropogenic challenges could struggle. While urbanization may favour a minority of species able to tolerate extreme human modification of habitat, even these species are subject to other human-caused threats that must be mitigated to ensure the sustained health of bird populations.

## Data Availability

Data have been deposited in the Open Science Framework: https://osf.io/cwp26/, (doi:10.17605/OSF.IO/CWP26) [50]. Supplementary material is available online [51].
